# Direct Bioelectrocatalytic Oxidation of Glucose by *Gluconobacter oxydans* Membrane Fractions in PEDOT:PSS/TEG-Modified Biosensors

**DOI:** 10.3390/bios11050144

**Published:** 2021-05-06

**Authors:** Anna Kitova, Sergei Tarasov, Yulia Plekhanova, Aleksandr Bykov, Anatoly Reshetilov

**Affiliations:** G.K. Skryabin Institute of Biochemistry and Physiology of Microorganisms, Pushchino Center for Biological Research of the Russian Academy of Sciences, Prosp. Nauki 5, Pushchino, 142290 Moscow, Russia; kitova@ibpm.pushchino.ru (A.K.); setar25@gmail.com (S.T.); agbykov@rambler.ru (A.B.)

**Keywords:** *Gluconobacter oxydans* membrane fractions, thermally expanded graphite, PEDOT:PSS, PEGDE, DMSO, direct bioelectrocatalytic oxidation, glucose biosensors

## Abstract

Recent years have witnessed an ever-increasing interest in developing electrochemical biosensors based on direct electron transfer-type bioelectrocatalysis. This work investigates the bioelectrocatalytic oxidation of glucose by membrane fractions of *Gluconobacter oxydans* cells on screen-printed electrodes modified with thermally expanded graphite and poly(3,4-ethylenedioxythiophene):poly(styrenesulfonate) (PEDOT:PSS). Electrooxidation of glucose was shown to occur without the presence of electron transport mediators. Chronoamperometric and cyclic voltametric characteristics showed an increase of anodic currents at electrode potentials of 0–500 mV relative to the reference electrode (Ag/AgCl). The direct electron transfer effect was observed for non-modified PEDOT:PSS as well as for PEDOT:PSS linked with crosslinkers and conductive fillers such as polyethylene glycol diglycidyl or dimethyl sulfoxide. Bioelectrodes with this composite can be successfully used in fast reagent-free glucose biosensors.

## 1. Introduction

The phenomenon of bioelectrocatalysis by the direct electron transfer (DET) between the active site of an enzyme and an electrode was first discovered and investigated in 1978. This effect was observed for laccase [1], peroxidase [2], and hydrogenase [3]. Mediatorless bioelectrocatalysis is widely used in such devices as biofuel cells and biosensors [4,5]. The biggest advantage of using this phenomenon is the minimization of bioelectrocatalytic reactions’ thermodynamic losses. Furthermore, there is no need for any additional reagents, so the bioelectrode design can be simplified significantly [6].

*Gluconobacter* bacteria contain membrane-bound dehydrogenases, which catalyze the oxidation of a number of lower alcohols and monosaccharides. The possibility of a DET from the active site of an enzyme to an electrode has been shown for some dehydrogenases isolated from these bacteria. Such dehydrogenases include aldehyde dehydrogenase [7], alcohol dehydrogenase [8], fructose dehydrogenase [9], gluconate dehydrogenase [10], and lactate dehydrogenase [11]. No DET effect has been observed for intact *Gluconobacter* cells. An alternative to the use of bacterial cells as biosensor biocatalysts is the use of their membrane fractions (MF), in which enzyme complexes are preserved in active state [12]. We demonstrated the use of this biocatalyst for creating a mediatorless alcohol biosensor [13]. Biosensors in greatest demand today are glucose detection devices [14], and *Gluconobacter oxydans* membrane fractions contain the pyrroloquinolinequinone(PQQ)-dependent glucose dehydrogenase [15]. For this reason, it seemed worthwhile to investigate the possibility of developing a DET-type glucose biosensor based on *G. oxydans* membrane fractions.

It should be noted that it is often necessary to use nanomaterials, such as carbon nanotubes [16], or modify the enzyme genetically [17,18], in order to achieve the DET effect. In [19], we used electrodes from a biocompatible carbon material, thermally expanded graphite (TEG). It is a graphene-like material that offers high electrical conductivity, large specific surface, and chemical stability [20]. The use of TEG as a modifier of the surface of standard screen-printed electrodes (SPE) would improve the electrochemical properties of biosensors for their real-world applications.

To achieve the DET effect, a conductive polymer is often required to ensure a tight contact between the electrode surface and the active site of the enzyme. The use of poly(3,4-ethylenedioxythiophene):poly(styrenesulfonate) (PEDOT:PSS) has been extensively studied in recent decades in bioelectronics and biomedicine [21,22]. PEDOT:PSS possesses a low oxidative potential and high conductivity [23]. In [24,25], a modification of enzyme biosensors for glucose detection with PEDOT:PSS and graphene made it possible not to use additional electron transfer mediators.

The aim of this work is to study the possibility of and conditions for the direct bioelectrocatalytic oxidation of glucose by membrane fractions of *G. oxydans* bacteria immobilized on SPE modified with TEG and PEDOT:PSS.

## 2. Materials and Methods

### 2.1. Reagents

Potassium phosphate dibasic trihydrate, sodium hydroxide, sodium chloride, urea, ascorbic acid (Mosreaktiv, Russia); potassium hexacyanoferrate(III), chitosan (low molecular weight), PEDOT:PSS (1.3 wt % dispersion in H_2_O), polyethylene glycol diglycidyl (PEGDE), dimethyl sulfoxide (DMSO) (Sigma, USA); sorbitol, glucose, yeast extract; bacteriological agar-agar, potassium chloride (Dia-M, Moscow, Russia) were used. Three-contact SPE were purchased from Color Electronics (Moscow, Russia). Whatman GF/A glass microfiber paper (Sigma, USA) was used to immobilize MF for Clark electrode measurements. As an electrode-modifying carbon material, we used TEG synthesized as described in [26].

### 2.2. Production of Gluconobacter oxydans Membrane Fractions

*Gluconobacter oxydans* sbsp. *industrius* VKM B-1280 (All-Russian Collection of Microorganisms) was used. Cells were grown in [27]. Membrane fractions of *G. oxydans* were produced by ultrasonic dispersion followed by step centrifugation as described in [28].

### 2.3. Preparation and Characterization of Biosensors

**Figure 1:**
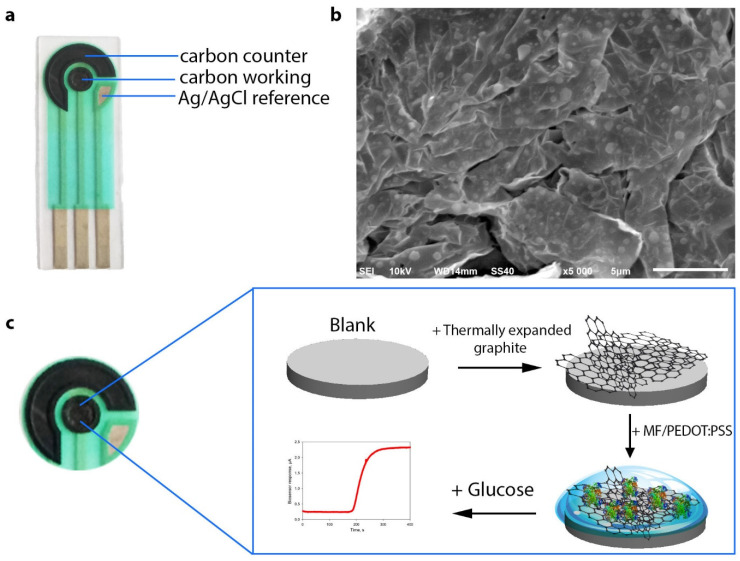
(**a**) Schematic of a graphite screen-printed electrode featuring counter, reference and working electrodes. (**b**) SEM image of thermally expanded graphite. (**c**) Schematic of the formation of a composite on the surface of the biosensor’s working electrode and its operating principle.

The screen-printed three-contact electrode consisted of a counter electrode, a working electrode formed from Electrodag 6017SS graphite paste (Henkel, Germany), and a reference electrode (Ag/AgCl) (Figure 1a). The working electrode was 3 mm in diameter. A layer of 0.1 mm TEG was formed on the working electrode by pressing at 150 Bar (Figure 1b). Membrane fractions of *G. oxydans* VKM B-11280 were immobilized on the working electrode surface using a 2% solution of chitosan in 1% acetic acid, a solution of PEDOT:PSS, as well as PEDOT:PSS solutions modified with 5% DMSO or 3% PEGDE (Figure 1c). The concentration of membrane fractions on the electrode surface was 0.4 mg/mm^2^. After applying the composite, the electrode was dried for 1 h at room temperature and then was left for 12 h at +4 °C.

Electrochemical measurements were carried out in a 2-mL cuvette at a temperature of 25 °C with constant stirring. As a background solution, we used a 25 mM potassium phosphate buffer (PBS), pH 6.5, containing 10 mM sodium chloride. All electrochemical measurements were conducted using IPCmicro (Kronas, Russia) and VersaSTAT 4 (Ametek, Berwyn, PA, USA) galvanostat potentiostats. The chronoamperometric curves were registered at an applied potential of +400 mV (vs. Ag/AgCl). The cyclic voltammograms (CVA) were registered at a potential scan rate of 3 mV/s within the range of 0 up to 500 mV. The impedance characteristics were measured in PBS at an applied potential of +200 mV (with 5 mM potassium ferricyanide) or +400 mV (without potassium ferricyanide) vs. Ag/AgCl within the range of frequencies from 40 kHz up to 0.02 Hz at a modulation amplitude of 10 mV.

The structure and morphology of the formed composites was examined by scanning electron microscopy (SEM) (JSM-6510LV 40, JEOL, Tokyo, Japan).

## 3. Results and Discussion

### 3.1. Optimization of Detection Electrodes

Screen-printed electrodes as the basis of electrochemical biosensors have a number of advantages over other types of electrodes—portability, speed of measurement, simplicity of use, low cost. For this reason, we used commercially accessible and cheap (<0.3€) carbon SPE that could be readily integrated into almost any measuring setup. Formation of the developed biosensor is shown in Figure 1c. The measuring setup consisted of a TEG/PEDOT:PSS-modified SPE connected to a potentiostat controlled by corresponding measuring software. Owing to the use of SPE, this system can be expanded for simultaneous monitoring of up to eight electrodes in real time mode by means of a multiplexer. Due to a modification of the graphite SPE surface with thermally expanded graphite, the active area of the biosensor surface increases to provide for a tighter contact of the biocatalyst with the electrode. The SEM image in Figure 1b shows that the TEG surface is uneven and has cavities for sorption of biological objects.

**Figure 2:**
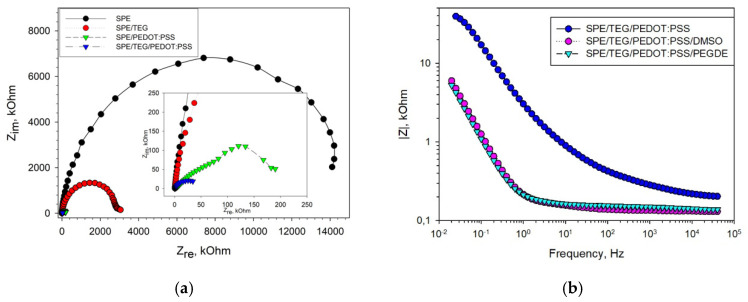
EIS Nyquist plots recorded in the presence of a 5 mM [Fe(CN)_6_]^3−^/^4−^ redox couple prepared in a 25 mM phosphate buffer with 0.01 M NaCl at an open-circuit potential (+200 mV vs. Ag/AgCl). (**a**) Change in impedance due to the presence of TEG, PEDOT:PSS and TEG/PEDOT:PSS on SPE; (**b**) change in the impedance profile due to a modification of PEDOT:PSS with DMSO and PEGDE.

A layer-by-layer application of components onto the surface of the working electrode was used. The effect of each of the successively applied components on the electrochemical properties of the electrode was evaluated by electrochemical impedance spectroscopy (EIS). Figure 2 presents Nyquist diagrams for non-modified SPE, as well as for SPE modified with TEG, PEDOT:PSS and their mixture. It is seen from the presented data that the use of TEG for modification of the graphite electrode surface not only increases its specific surface area, but also significantly decreases the charge-transfer resistance and the total impedance of the electrode, which subsequently improves the electrical conductivity between the active site of the enzyme and the electrode surface. PEDOT:PSS has a mixed electron–ion type of conductivity and also reduces the impedance of the graphite electrode. The lowest total resistance (*R* = 49 kOhm) was achieved when using an SPE/TEG/PEDOT:PSS composite. Besides, Figure 2b shows EIS spectra for an SPE/TEG/PEDOT:PSS composite at an additional modification of PEDOT:PSS with DMSO and PEGDE. Nyquist diagrams for those EIS spectra are presented in Figure S1; a Randles equivalent circuit used to fit the impedance data is presented in Figure S2a. The use of various solvents [29] to improve the conductivity of PEDOT:PSS gel was described. PEGDE interacts with PSS chains to form a three-dimensional highly conductive network. At the same time, addition of DMSO leads to a redistribution of conductive PEDOT particles and the removal of excess PSS particles from the surface, which establishes an easier way for charge transfer into the polymer film, thus increasing the conductivity of the system. It is clearly seen from the data obtained that both compounds greatly reduce the impedance of the electrode with PEDOT:PSS within the entire range of investigated frequencies.

### 3.2. Respiratory Activity of G. oxydans Membrane Fractions

**Figure 3:**
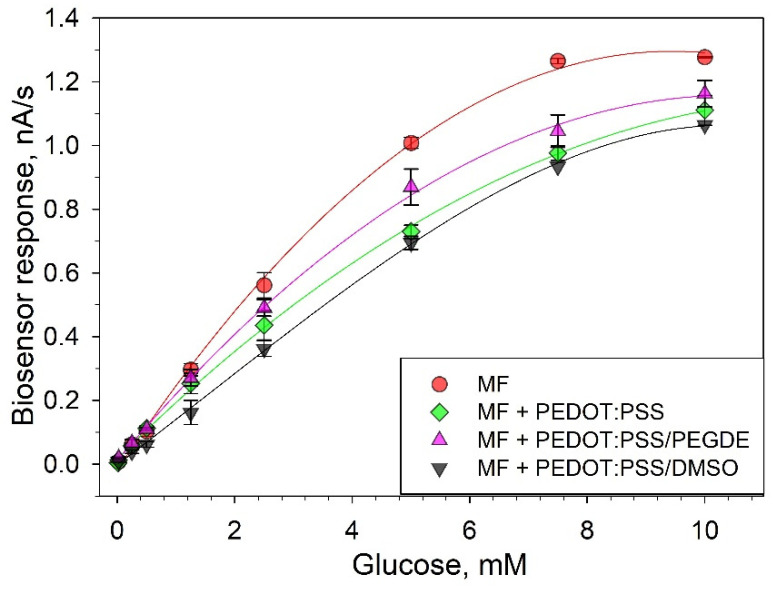
Respiratory activity of membrane fractions in the presence and absence of PEDOT:PSS gels.

As membrane fractions of *G. oxydans* bacteria represent fragments of the respiratory chain, we used a Clark-type oxygen electrode to characterize their catalytic activity in the glucose oxidation reaction. To determine the extent of the possible negative effect of the system’s components on the catalytic activity of the respiratory chain enzymes, we applied a mixture of membrane fractions and PEDOT:PSS gel with various modifications onto a fragment of Whatman GF/A, 3 × 3 mm^2^ in size. This fragment was then used as a bioreceptor for the Clark-type oxygen electrode. The rate of the bioreceptor’s respiratory activity variation did not practically change depending on the concentration of MFs on the electrode surface within the range of 0.02–0.1 mg/mm^2^ (Figure S3). The effect of polymer gel on the activity of the respiratory chain enzymes was determined by the change of the parameters of the biosensor’s calibration curve presented in Figure 3.

The respiratory activity of membrane fractions decreased in the presence of all variants of PEDOT:PSS gels. On the linear segment of the calibration curve, a decrease of the biosensor signal was 25% for PEDOT:PSS, 14% for PEDOT:PSS/PEGDE and 37% for PEDOT:PSS/DMSO. The affinity of an enzyme for its substrate was also evaluated using the apparent Michaelis–Menten constant, *K*_M_. When PEDOT:PSS was introduced into the system, the constants increased from 3.56 (for a system without polymer gel) to 6.91 (for a PEDOT:PSS/DMSO system). This implies that the used polymers decrease the affinity of the enzyme complexes of the *G. oxydans* respiratory chain to glucose. Herewith, the addition of an extra stabilizing agent PEGDE to PEDOT:PSS led to a decrease of the said negative effect, which once again proves the efficiency of using additional PEDOT:PSS-modifying agents in bioelectrodes.

### 3.3. Electrochemical Parameters of Bioelectrodes

**Figure 4:**
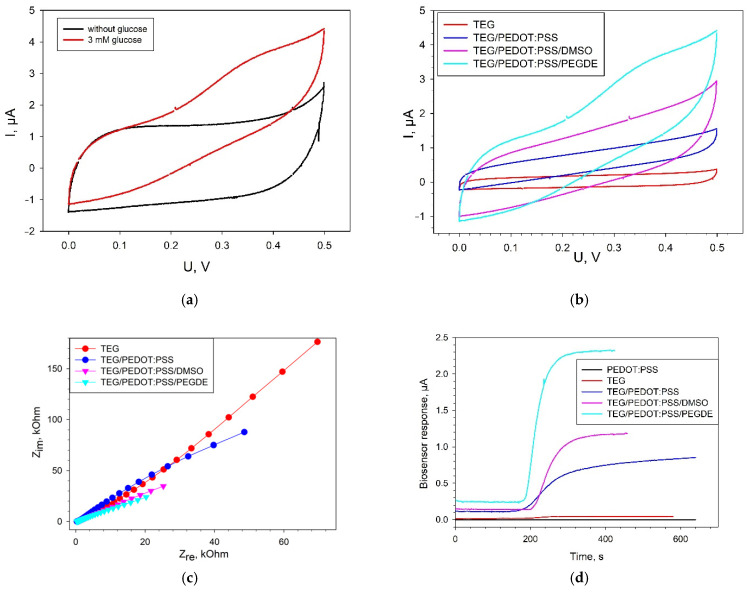
Electrochemical characteristics of biosensors modified with various types of PEDOT:PSS. (**a**) CVA of a TEG/PEDOT:PSS/PEGDE bioelectrode in the presence and absence of 3 mM glucose at a scan rate of 3 mV s^−1^; (**b**) CVA of various modifications of bioelectrodes in the presence of 3 mM glucose at a scan rate of 3 mV s^−1^; (**c**) Nyquist plots for all types of bioelectrodes; (**d**) biosensor signals in response to the addition of 3 mM glucose at an applied potential of +400 mV (vs Ag/AgCl). All measurements were made without the presence of redox mediators.

The electrochemical characteristics of the produced composites based on PEDOT:PSS and membrane fractions were investigated by cyclic voltammetry, chronoamperometry, and electrochemical impedance spectroscopy. Figure 4a presents cyclic voltammograms for a bioelectrode with a TEG/PEDOT:PSS composite in the presence and absence of glucose. Membrane fractions immobilized on the electrode surface in PEDOT:PSS gel interact directly with the electrode in the presence of glucose, which is expressed in an increase of anodic currents within the range of 0.1 up to 0.5 V. Electroactive enzymes present in membrane fractions transfer the electrons formed in the oxidation of glucose to the electrode without the presence of any additional reagents. Most likely, PQQ, which is already present in the membrane fractions of the bacteria [30], acts as an electron carrier. The discrepancy between voltammograms in the presence or absence of glucose was observed for all types of PEDOT:PSS. The maximal discrepancy between voltammograms in the presence and absence of glucose was observed for a TEG/PEDOT:PSS/PEGDE composite.

Figure 4b presents the cyclic voltammograms for four types of bioelectrodes in the presence of 3 mM glucose. All reported CVA curves for electrodes containing PEDOT:PSS (Figure 4b) were of rectangular shape, which is typical of PEDOT in an aqueous medium and is indicative of a capacitive behavior of these materials. The maximal level of anodic currents within the range of 0 up to 0.5 V was obtained for the bioelectrode with a TEG/PEDOT:PSS/PEGDE composite.

When choosing the applied potential for the biosensor, we compared the analytical performance of the bioelectrode at different applied potentials. The chronoamperometric curves for these applied potentials are shown in Figure S4. Since the maximum signal upon addition of 0.5 mM glucose was observed at an applied potential of 400 mV, we used this applied potential to obtain EIS spectra and chronoamperometric curves.

The EIS spectra of the investigated composites feature a decrease of impedance of the system in the sequence TEG > TEG/PEDOT:PSS > TEG/PEDOT:PSS/DMSO > TEG/PEDOT:PSS/PEGDE (Figure 4c). This is consistent with the data obtained for the same composites without the biocatalyst (Figure 2b). It is to be noted that the impedance of electrodes with membrane fractions in the presence of substrates is generally lower than for electrodes without membrane fractions. This is indicative of an electron transfer from the enzyme systems to the electrode in the absence of an additional electron-transport mediator. Simulations of the EIS data using an equivalent circuit model (modified Randles equivalent circuit, Figure S2b) showed that the value of charge transfer resistance was 426 kOhm for a TEG bioelectrode; 143 kOhm for TEG/PEDOT:PSS; 49 kOhm for TEG/PEDOT:PSS/DMSO, and 33 kOhm for TEG/PEDOT:PSS/PEGDE.

Typical signals for an amperometric biosensor in response to the addition of glucose are given in Figure 4d. A bioelectrode, the surface of which was not modified with TEG but only with PEDOT:PSS gel, yielded no signal to glucose addition. Herewith, it should be noted that even in the absence of PEDOT:PSS, at the electrode modified with TEG, we observed the transfer of electrons from the enzyme active site to the electrode at the transformation of substrate. The amplitude of the signal for TEG-modified electrode upon addition of 3 mM glucose was 23 ± 4 nA. For biosensors modified with PEDOT:PSS, the amplitude of the signal upon addition of glucose increased from 23 ± 4 nA (TEG) up to 0.74 ± 0.04 µА (TEG/PEDOT:PSS), 1.04 ± 0.05 µА (TEG/PEDOT:PSS/DMSO) and 1.97 ± 0.18 µА (TEG/PEDOT:PSS/PEGDE). Thus, according to all electrochemical methods, the TEG/PEDOT:PSS/PEGDE composite outperforms the other PEDOT:PSS formulations.

**Figure 5:**
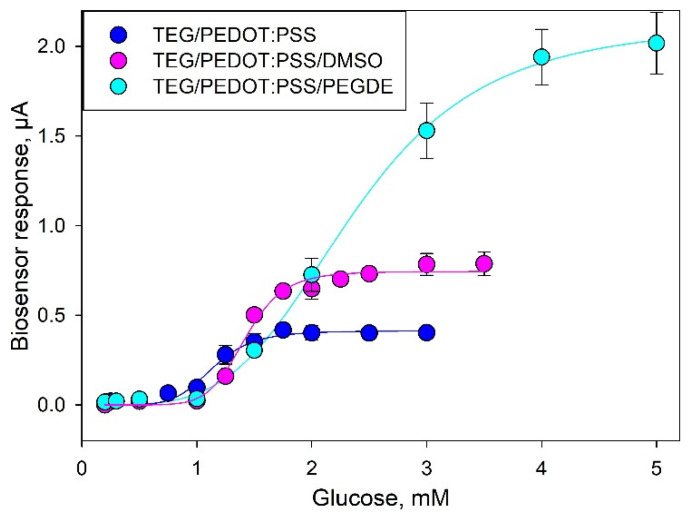
Calibration curves for glucose (values averaged from three consecutive calibrations).

In order to assess the analytical performance of the proposed biosensors, we plotted calibration curves of current vs. glucose concentration (Figure 5). The analytical parameters for the three types of biosensors are presented in Table 1. The data from the glucose calibration curves were analyzed using the Michaelis–Menten kinetics with the measured current serving as the reaction velocity [31]:

(1) $$I=\frac{I_{max}S^{h}}{K_{M}^{h}+S^{h}}$$

Comparing the metrological characteristics of the developed systems and their analogues, it can be seen that the lower limits of the linear ranges of detection for all three composites are approximately the same. The amplitude of the signal upon addition of glucose and the maximum achievable current (*I_max_*) increase when using modified variants of composites. Moreover, the biosensor sensitivity coefficient at an additional modification of PEDOT:PSS with DMSO or PEGDE increased from 0.46 up to 0.8 or 0.82 μA/mM. The apparent Michaelis constant (K_m_), which is the substrate concentration needed to achieve half of the *I_max_* value, increases in the sequence TEG/PEDOT:PSS > TEG/PEDOT:PSS/DMSO > TEG/PEDOT:PSS/PEGDE. The Hill coefficient (*h*) is a dimensionless value that characterizes the cooperativity of ligand binding by the enzyme. As seen from the data obtained, in all cases, the Hill coefficient is greater than 1, i.e., a positive cooperativity of the enzymes is observed for MFs immobilized in all types of PEDOT:PSS polymers. It should also be noted that the single measurement time decreased to 2 min when using a TEG/PEDOT:PSS/PEGDE composite, which is due to a decrease of electrode impedance and an increase of electron transfer rate in the system.

**Figure 6:**
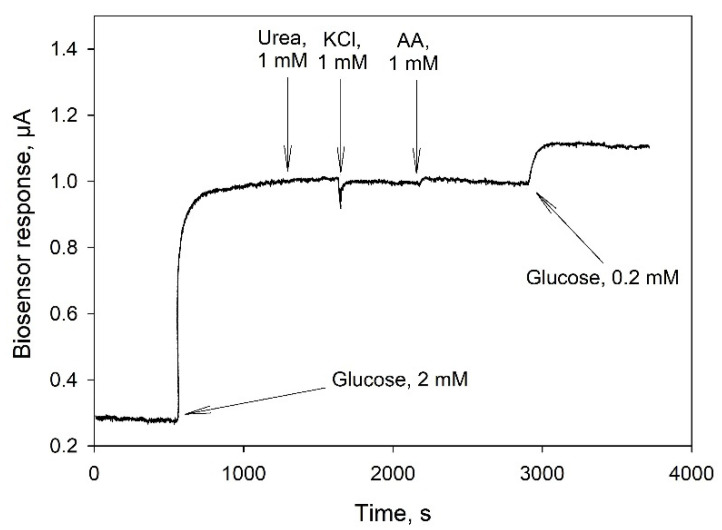
Amperometric response of a TEG/PEDOT:PSS/PEGDE biosensor to the addition of glucose and some interferents at a potential of 0.4 V (vs Ag/AgCl).

In biosensor applications, a key concern is the fact that the selectivity of a glucose sensing device can be decreased by interference from some coexisting substances in real samples. As membrane fractions of bacteria contain a whole range of enzyme complexes, it was necessary to understand which substances could hinder the accurate determination of glucose in real blood samples. Figure 6 presents an amperometric signal of a biosensor based on TEG/PEDOT:PSS/PEGDE in response to some potential interferents including urea, ascorbic acid (AA) and KCl in an oxygen-containing PBS solution (0.1 M, pH 6.5) under an operating potential of 0.4 V. None of the above interferents evoked any notable response from the biosensor for 1500 s after their introduction, while the addition of 2 mM glucose caused a significant increase (0.7 µA) of the current level within 100 s. Thus, the membrane fraction/TEG/PEDOT:PSS/PEGDE electrode exhibited a good selectivity towards glucose over these molecules that can be found in the human blood.

## 4. Conclusions

In this work, we investigated mediatorless bioelectrocatalytic oxidation of glucose by *G. oxydans* membrane fractions on SPE modified with TEG. Membrane fractions were immobilized on screen-printed TEG-modified electrodes into PEDOT:PSS-conductive polymer, as well as PEDOT:PSS activated with PEDGE or DMSO. The electrochemical reaction was shown to occur in the absence of artificial redox mediators. Introduction of additional reagents, such as DMSO and PEDGE, led to a decrease of the electrode charge transfer resistance, a decrease of the negative effect of polymer on the biocatalyst’s respiratory activity, an increase of the biosensor signal amplitude, and an increase of its sensitivity to glucose. Besides, the use of TEG/PEDOT:PSS/PEGDE composite enabled reducing the glucose single assay time to 2 min as compared with 5–6 min for TEG/PEDOT:PSS. One of the additional advantages of the developed biosensor is that the costly protein purification step can be skipped, and stability can be greatly improved. This indicates that these biosensors have a great potential for clinical application. The presented strategy can also provide insights into the development of other biosensors or biofuel cells based on membrane fractions of bacterial cells.

## Figures and Tables

**Table 1:** Analytical characteristics of PEDOT:PSS biosensors for glucose.

	Modification	TEG/PEDOT:PSS	TEG/PEDOT:PSS/DMSO	TEG/PEDOT:PSS/PEGDE
Parameter				
*I*, μА		0.41	0.74	2.13
*K*_m_, mM		1.14	1.42	2.36
*h*		6.48	8.48	4.08
Linear range of detection, mM		0.81–1.59	0.90–1.90	1.03–3.01
Regression equation for the linear segment		*y* = 0.46*x* − 0.33	*y* = 0.80*x* − 0.77	*y* = 0.82*x* − 0.89
Correlation coefficient, *R*^2^		0.98	0.98	0.99
Sensitivity coefficient, μA/mM		0.46	0.80	0.82
Single measurement time, min		5–6	4–5	2–2.5

The correlation coefficient for the calibration curves and for the regression equation for the linear segment of *R*^2^ is 0.98.

## Data Availability

Not applicable.

## Supplementary Materials

The following are available online at https://www.mdpi.com/article/10.3390/bios11050144/s1, Figure S1: Change in EIS Nyquist plots, recorded in the presence of a 5 mM [Fe(CN)_6_]^3−^/^4−^ redox couple prepared in a 25 mM phosphate buffer with 0.01 M NaCl at an open-circuit potential (+200 mV vs. Ag/AgCl) due to the presence of TEG/PEDOT:PSS, TEG/PEDOT:PSS/DMSO and TEG/PEDOT:PSS/PEGDE on SPE, Figure S2: Randles equivalent circuit (**a**) and modified Randles equivalent circuit (**b**) used to fit Nyquist plots for electrodes. Rs, solution resistance; Rct, charge-transfer resistance; Cdl, double-layer capacitance; Zw, Warburg impedance, Figure S3: Respiratory activity of membrane fractions in the presence of glucose (0.5 mM) as a function of their concentration in a bioreceptor, Figure S4: TEG/PEDOT:PSS/PEGDE biosensor signals in response to the addition of 0.5 mM glucose at various applied potentials (vs Ag/AgCl) without the presence of redox mediators.
