# Supplementary material for: Direct Bioelectrocatalytic Oxidation of Glucose by Gluconobacter oxydans Membrane Fractions in PEDOT:PSS/TEG-Modified Biosensors
Source: Biosensors (Basel). 2021 May 6;11(5):144. doi: 10.3390/bios11050144 (PMC8148135; doi:10.3390/bios11050144)
Supplement: Supplementary file 1 [file biosensors-11-00144-s001.zip › biosensors-1186472-supplementary.pdf]

biosensors

MDPI

Supplementary

Direct Bioelectrocatalytic Oxidation of Glucose by *Gluconobacter oxydans* Membrane Fractions in PEDOT:PSS/TEG-Modified Biosensors

Anna Kitova, Sergei Tarasov, Yulia Plekhanova *, Aleksandr Bykov and Anatoly Reshetilov *

G.K. Skryabin Institute of Biochemistry and Physiology of Microorganisms, Pushchino Centre for Biological Research, Russian Academy of Sciences, 5 Prosp. Nauki, Pushchino, Moscow Region, 142290, Russian Federation; kitova@ibpm.pushchino.ru (A.K.); setar25@gmail.com (S.T.); agbykov@rambler.ru (A.B.)

* Correspondence: plekhanova@ibpm.pushchino.ru (Y.P.); anatol@ibpm.pushchino.ru (A.R.)

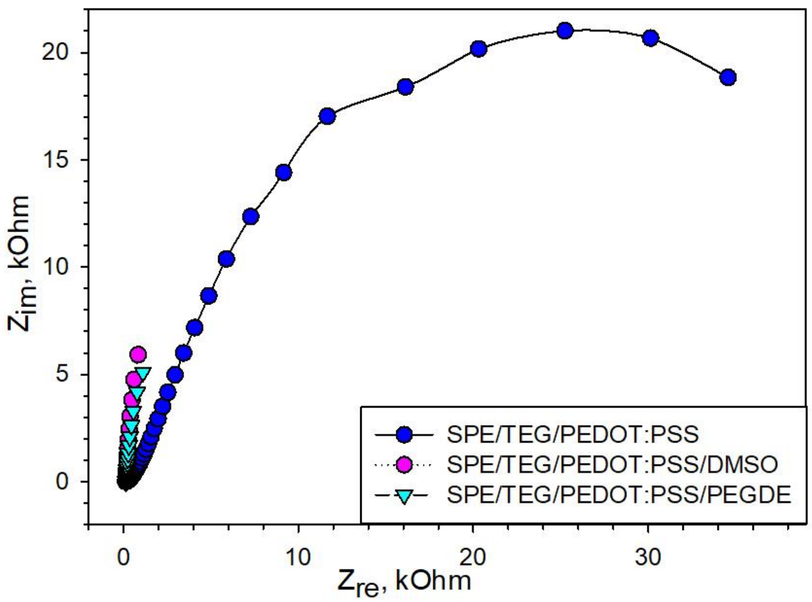

Figure S1. Change in EIS Nyquist plots, recorded in the presence of a 5 mM $[Fe(CN)_6]^{3-/4-}$ redox couple prepared in a 25 mM phosphate buffer with 0.01 M NaCl at an open-circuit potential (+ 200 mV vs. Ag/AgCl) due to the presence of TEG/PEDOT:PSS, TEG/PEDOT:PSS/DMSO and TEG/PEDOT:PSS/PEGDE on SPE.

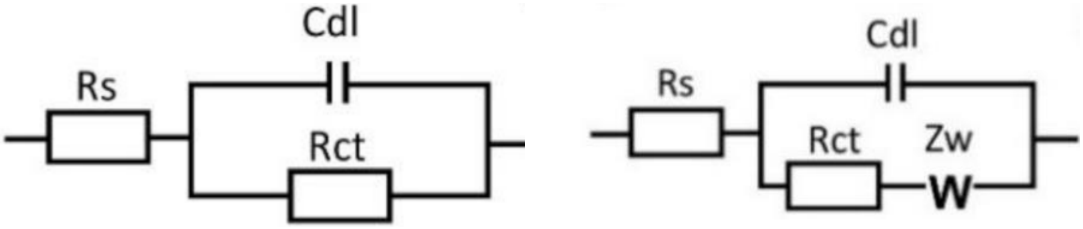

(**a**) (**b**)

Figure S2. Randles equivalent circuit (**a**) and modified Randles equivalent circuit (**b**) used to fit Nyquist plots for electrodes. Rs, solution resistance; Rct, charge-transfer resistance; Cdl, double-layer capacitance; Zw, Warburg impedance.

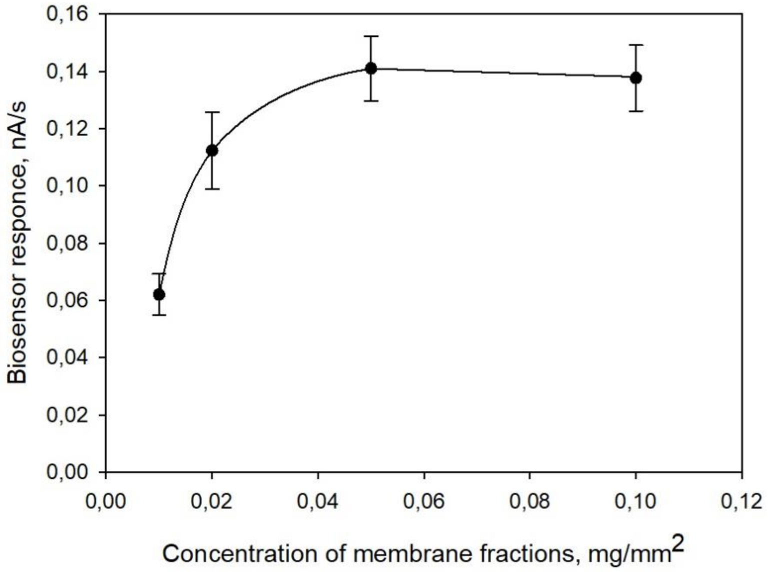

Figure S3. Respiratory activity of membrane fractions in the presence of glucose (0.5 mM) as a function of their concentration in a bioreceptor.

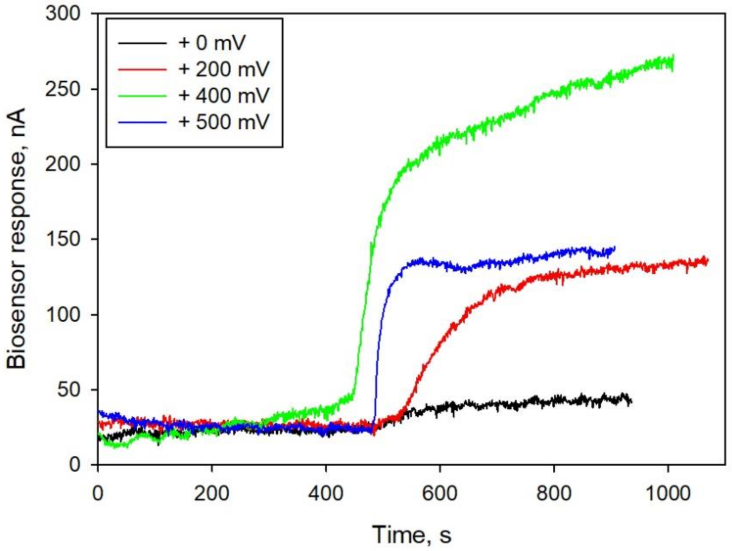

Figure S4. TEG/PEDOT:PSS/PEGDE biosensor signals in response to the addition of 0.5 mM glucose at various applied potentials (vs Ag/AgCl) without the presence of redox mediators.
